# Naturalism and the hard problem of mysticism in psychedelic science

**DOI:** 10.3389/fpsyg.2024.1112103

**Published:** 2024-03-15

**Authors:** Jussi Jylkkä

**Affiliations:** Department of Psychology, Åbo Akademi University, Turku, Finland

**Keywords:** psychedelics, mystical experience, naturalism, physicalism, hard problem of consciousness, metaphysics

## Abstract

Psychedelic substances are known to facilitate mystical-type experiences which can include metaphysical beliefs about the fundamental nature of reality. Such insights have been criticized as being incompatible with naturalism and therefore false. This leads to two problems. The easy problem is to elaborate on what is meant by the “fundamental nature of reality,” and whether mystical-type conceptions of it are compatible with naturalism. The hard problem is to show how mystical-type insights, which from the naturalistic perspective are brain processes, could afford insight into the nature of reality beyond the brain. I argue that naturalism is less restrictive than commonly assumed, allowing that reality can be more than what science can convey. I propose that what the mystic refers to as the ultimate nature of reality can be considered as its representation- and observation-independent nature, and that mystical-type conceptions of it can be compatible with science. However, showing why the claims of the mystic would be true requires answering the hard problem. I argue that we can in fact directly know the fundamental nature of one specific part of reality, namely our own consciousness. Psychedelics may amplify our awareness of what consciousness is in itself, beyond our conceptual models about it. Moreover, psychedelics may aid us to become aware of the limits of our models of reality. However, it is far from clear how mystical-type experience could afford access to the fundamental nature of reality at large, beyond one’s individual consciousness. I conclude that mystical-type conceptions about reality may be compatible with naturalism, but not verifiable.

## 1 Introduction

Psychedelic substances^1^ are known to facilitate mystical-type experiences, which may include metaphysical insights about the fundamental nature of reality, not attainable by the senses or intellect^2^. Such insights could be expressed by saying that “All is One,” or that the fundamental nature of reality is, as Ram Dass puts it, “loving awareness,” or even something that could be referred to as “God.” Typically, such insights are considered to reveal the nature of reality at large, not just one’s own individual consciousness. Some naturalistically oriented scientists and philosophers might consider the insights as unscientific and therefore false. For example, a prominent philosopher of psychedelics, Letheby (2021), considers mystical-type metaphysical insights as inconsistent with naturalism and sees them as *negative side-effects* of psychedelic experiences, or metaphysical hallucinations. In a recent commentary paper, Sanders and Zijlmans (2021) considered the mystical experience as the “elephant in the living room of psychedelic science” (p. 1253) and call for the demystification of the field. Carhart-Harris and Friston (2019), following Masters (2010), refer to spiritual-type features of psychedelic experiences as *spiritual bypassing*, where one uses spiritual beliefs to avoid painful feelings, or “what really matters.” While this may be true in some cases, it certainly is not always.

In contrast to the naturalistic researchers cited above, the advocates of the *mystical approach* would hold that, at least some types of psychedelically facilitated metaphysical insights can be true. For example, a prominent developer of psychedelic-assisted therapy, psychologist Bill Richards holds that psychedelics can yield “sacred knowledge” not afforded by the typical means of perception and rational thinking, and which can have therapeutic potential (Richards, 2016). The eminent religious scholar Huston Smith holds that “the basic message of the entheogens [is] that there is another Reality that puts this one in the shade” (Smith, 2000, p. 133). Several contemporary philosophers are taking the mystical experiences seriously and aim to give them consistent conceptualizations. For example, Peter Sjöstedt-Hughes has interpreted experiences facilitated by the psychedelic substance 5-MeO-DMT, characterized by an experience of unitary white light that underlies the perceptual reality, in terms of Spinoza’s philosophy, where it could be considered to reveal the ultimate nature of reality, which for Spinoza is equal to God (Sjöstedt-H, 2022). Likewise, Steve Odin, a philosopher who specializes in Buddhist philosophy, argues that LSD-induced experiences may promote a *satori* experience where one can be considered to become acquainted with the *dharmakāya*, or the Buddha-nature of reality (Odin, 2022). I have also argued previously that unitary experiences, which can be facilitated by psychedelics, enable us to know what consciousness is in itself, thereby yielding *unitary knowledge* which is unlike *relational knowledge* afforded by perception and other modes of representation (Jylkkä, 2022). These authors continue a long tradition in perennialistic psychedelic science, defended by key figures like James (1902), Huxley (1954), and Watts (1962) where mystical experiences are taken to reflect a culture-independent common core, which can reveal us the “Reality of the Unseen” (to borrow a phrase from James).

From the neuroscientific perspective, a mystical-type experience is just like any other experience, that is, a biochemical process in the brain inside the skull. The subject undergoing a psychedelic experience in a functional magnetic resonance imaging device (fMRI) during a scientific experiment does not become dissolved in their environment, or at least so it appears. What the mystic considers as an ineffable revelation of the fundamental nature of reality, the neuroscientist considers as a brain process. The problem is, then: why should the brain process tell the mystic anything of reality outside the skull? Mystical experience is, after all, unlike sense perception where the perceiver is causally linked with the perceived, external object. In mystical experience, the mystic is directed inwards and is not, at least so it seems, basing their insight on any reliable causal interaction with the reality at large. The mystic’s insight is not verifiable in the same sense as empirical observation. Thus, how could the mystical experience yield knowledge of reality at large, instead of just their own individual consciousness? This can be considered as the *hard problem* of mysticism. Another problem pertains to the *compatibility* between the mystic’s claims about reality. For example, when the mystic claims that God is the fundamental nature of reality, is this compatible with what we know about the world through science? (In this paper, by “science” I refer to natural science, unless states otherwise.) Answering this question requires elaborating on what is meant by the “ultimate nature of reality,” and whether that notion is compatible with naturalism. We may call this the *easy problem* of mysticism.^3^ I will argue that the easy problem may be solvable: it could be compatible with naturalism to hold that there is an ultimate nature of reality unknown to science, and some mystical-type claims about that ultimate nature may be compatible with naturalism. However, this compatibility does not entail that the mystical-type claims about reality would be *true*. This leads to the hard problem: What could be the epistemic mechanism that renders the mystical-type claims about reality true?

I will first focus on the easy problem about the compatibility between mysticism and naturalism. I examine Letheby’s (2021) argument that mystical-type metaphysical insights (or, more specifically, their conceptualizations) are incompatible with naturalism, focusing on the concept of naturalism. I argue that naturalism is more liberal than Letheby assumes, and that naturalism is not very restrictive about what can be considered as “natural”; this can be considered as an *a posteriori* question. Moreover, I argue that naturalism allows there to be more ways of knowing nature than just science, unless naturalism is conflated with scientism. In other words, there can be more to knowledge than science can confer. The limits of science are illustrated with the case of consciousness, which can for good reasons be considered as a physical process, but which nevertheless cannot be fully conveyed by science: from science we cannot infer what it is like to be a bat, to experience colors, or to undergo a psychedelic experience. I propose that science cannot fully capture the intrinsic nature of consciousness, because it cannot fully capture the intrinsic nature of *anything* – this is a general, categorical limit of science. Science is limited to modeling the world based on observations and “pointer readings” but cannot convey what is the model-independent nature of the modeled, that is, the nature of the world beyond our representations of it. This representation-independent nature of reality can be considered as its “ultimate nature,” which can be represented in several ways. This opens up the possibility that mystical-type claims about reality could be true, or at least not ruled out by the scientific worldview. The scientific worldview is, after all, just a *view* of reality, and there can be several ways to represent reality. I will then turn to the hard problem, arguing that there is a case where we can directly know the ultimate nature of reality, and that is the case of our own consciousness. I know my consciousness directly through *being* it, not merely through representing it. This type of knowledge can be called *unitary*, in contrast to representational or observational knowledge, which is *relational*. Consciousness can be argued to directly reveal the ultimate nature of one specific form of the physical reality, namely that of those physical processes that constitute human consciousness. This, however, leaves open the hard problem: how could the mystic know the nature of reality at large through their own, subjective experience? What is it about the mystical-type experience that could afford the mystic insight into the nature of reality at large? I will conclude by examining some possible approaches to the hard problem.

## 2 Metaphysical insights in mystical-type experiences

To evaluate whether metaphysical insights involved in mystical experiences are compatible with naturalism, we must first examine how they are typically conceptualized. Current research on mystical-type experiences in the psychedelic context is largely based on the work of the philosopher Stace (1961). In the empirical context, mystical-type experiences are commonly assessed with the Mystical Experience Questionnaire (MEQ) (Barrett et al., 2015), which is probably the single most important predictor of treatment outcomes in psychedelic-assisted therapy (Ko et al., 2022; but see Letheby, 2021). The MEQ is based on Stace’s work, that is, it aims to assess mystical-type experience as originally defined by Stace. This motivates focusing on Stace’s theoretical work in the present philosophical discussion of mystical-type experiences and their metaphysical features.

Drawing from historical mystics across epochs and cultures, Stace identifies the following universal core features of the mystical experience: (1) The experience includes a strong sense of unity with the environment or the sense that “All is One,” and at least in some varieties (the so-called internal unitary experience) it has no sensory content. (2) The experience is perceived as non-spatial and nontemporal and may thus be experienced as infinite or outside time. (3) It has a sense of objectivity or reality, or what William James called “noetic quality,” meaning that it is felt as true or as revealing something that is true. (4) It involves feelings of blessedness and joy, which may be considered as not merely subjective experiences, but rather as stemming from contact with the ultimate nature of reality. (5) The experiencer senses that they have met something divine or sacred. (6) The experience involves paradoxical aspects, such as perceiving unity in individual objects and the many in One, yielding a logical contradiction. Finally, (7) the experience is alleged by the experiencer to be ineffable or impossible to capture in words (Stace, 1961, 110–111).

Stace’s original list of the features of mystical experiences contains at least two clear metaphysical insights: that of unity, and that of being outside time and space.^4^ Depending on the case, the sense of blessedness and joy could also count as metaphysical insights if the person experiences them to reflect the fundamental nature of reality (e.g., that the fundamental nature of reality is somehow sacred or divine, or otherwise intrinsically positively valued). Also, the notions of paradoxicality and ineffability could be counted as metaphysical insights to the extent that they imply that the fundamental nature of reality cannot be captured in language. Noetic quality might appear as metaphysical, but it is mainly a second-order feature that pertains to the epistemic status of the insight (i.e., it is felt as true), not its content. Nevertheless, possessing noetic quality is arguably a necessary condition for an experience to count as “metaphysical insight” – the experience cannot be a metaphysical insight unless it is perceived as true.

Unity is considered by Stace to be the most central feature of the mystical-type experience. He divides the experience of unity into internal and external. The former means an empty consciousness void of any sensory contents that may nevertheless be characterized as “light” or “consciousness,” as is done by Jan van Ruysbroeck here:

The God-seeing man … can always enter, naked and unencumbered with images, into the inmost part of his spirit. There he finds revealed an Eternal Light … It [his spirit] is undifferentiated and without distinction, and therefore it feels nothing but the unity (Quoted in Stace, 1961, p. 94).

A similar insight was experienced by the theologist-physician-psychiatrist Walter Pahnke, renowned for his contributions in the early stages of psychedelic science, during his first LSD trip:

The most impressive and intensive part of this experience was the *white light* of absolute purity and cleanness … The associated feelings were those of absolute *awe*, *reverence* and *sacredness* … The white light experience was of *supreme importance* – absolutely self validating and something worth staking your life on and putting your trust in (Pahnke, 1964).^5^

It is obvious from the quote that Pahnke did not consider the experience as *just* an experience, but rather as something truly existing that one can “put their trust in”; thus, it had noetic quality and can be considered as a metaphysical insight. The absoluteness and all-encompassing reality of such “illumination” is also emphasized in, for example, the teaching of the Zen master Huangbo, who calls it the “One Mind” or simply “Buddha”:

All the Buddhas and all sentient beings are nothing but the One Mind, beside which nothing exists. The One Mind alone is the Buddha, and there is no distinction between the Buddha and sentient beings (Quoted in Blofeld, 1958).

External unity, in turn, means an experience where the subject experiences the unity in the multiplicity of external objects. For example, St Teresa expresses her experience as follows:

One day being in orison it was granted to me to perceive in one instant how all things are seen and contained in God. I did not perceive them in their proper form, and nevertheless the view I had of them was of a sovereign clearness, and has remained vividly impressed upon my soul (Quoted in Stace, 1961, p. 68).

In experiences of external unity, one typically sees every single object as an instance of one single underlying reality, which supersedes that which can be perceived with the senses. St Teresa calls it “God,” and Meister Eckhart calls it the “One”:

Here all blades of grass, wood, and stone, all things are One. This is the deepest depth (Quoted in Stace, 1961, p. 63).

How is it that the mystic knows these things? The mystical insight pertains to the nature of reality that cannot be known through the senses or the intellect (i.e., conceptual-rational thinking), but rather which can only be directly intuited, or known through becoming one with it. This is prominent in typical definition of “mystical,” as for example in the Merriam-Webster dictionary:

Having a spiritual meaning or reality that is neither apparent to the senses nor obvious to the intelligence (Merriam-Webster, n.d.)

Or as:

Involving or having the nature of an individual’s direct subjective communion with God or ultimate reality. (*Ibid.*)

Here the first definition pertains to what the mystical *is*, while the latter definition is about how a subject can *know* the mystical: through direct communion or becoming one with it. Importantly, the definitions do not make positive characterizations about the nature of that something that one has encountered in a mystical experience (although it is often referred to by mystics as “God” or “ultimate reality”). This could be taken to reflect the intrinsic ineffability or non-conceptual nature of the insight, to which all conceptualizations are subordinate (Stace, 1961).

To sum up, we can characterize the metaphysical-epistemological Core of the mystical experience as follows:

(Core) There is a fundamental, unitary nature of reality that is beyond the sensory world and that one can know directly.

Note that the Core contains both a metaphysical element that refers to the ultimate nature of reality, and an epistemological element implying that we can know that nature directly. The Core does not imply what the fundamental nature of reality *is*, and arguably different people can have different conceptions about it, although in both classical mystics and psychedelic reports notions like “Light” or “Mind” occur repeatedly. The easy problem mainly pertains to the metaphysical part of the thesis and includes the following questions:

(EP1) Can we reconcile with science the notion that there is a unitary, fundamental nature of reality that is beyond the scope of observation-based science?

(EP2) To what extent are different mystical-type theses of this alleged “fundamental nature” compatible with science?^6^

I will mainly focus on the first question, since answering it is a prerequisite for answering the second one. The hard problem, in turn, pertains mainly to the epistemic part of the Core and can be summarized as follows:

(HP) How could the mystical-type insight give access to the unitary, fundamental nature of reality in a direct, non-sensory and non-intellectual sense?

The hard problem involves showing what is the relationship between the mystical-type insight and the nature of reality at large, beyond the mystic’s own consciousness. Moreover, there is a more specific problem pertaining to the relationship between mystical-type *conceptions* of reality (e.g., that the ultimate nature of reality is God, love, or a cosmic consciousness) and the mystical-type *insight*, which is arguably non-conceptual and non-representative. The mystical-type insight is often considered as ineffable and direct, leading to the question of how it can justify or ground conceptual representations of reality. In sum, the hard problem involves showing how the mystical-type insight could directly reveal the ultimate nature of reality, as well as how it could justify specific mystical-type theses of reality. Here I am mainly concerned with the first part of the hard problem, that is, how the insight could reveal reality at large.

Next, I will focus on the easy part by examining what is meant by naturalism. I will argue that naturalism, as commonly conceived, is compatible with the existence of non-scientific knowledge, and with the idea that there is an ultimate nature of reality beyond the scope of science.

## 3 Naturalism

Naturalism has no agreed meaning and only few contemporary philosophers would consider themselves as non-naturalists (Papineau, 2020). Nevertheless, it is commonly accepted that naturalism consists of two main components, ontological and epistemological. The ontological component is commonly equated with physicalism, the metaphysical thesis that only physical entities or processes exist (Papineau, 2020). I will follow this tradition. The epistemological component, in turn, is more difficult to pinpoint. It is commonly taken to imply that our primary means of knowing reality are scientific, or that philosophy should in some sense be continuous with the sciences.

For example, in *The Concise Encyclopedia of Western Philosophy and Philosophers*, edited by Urmson and Ree, naturalism is defined as follows:

A naturalist considers that the totality of all things which we call ‘nature’ and which are studied in the natural sciences is the totality of all things whatever, and denies the need of any explanations of the natural in terms of the super-natural; such a philosopher will normally hold that any reference to a deity, or to a realm of values, or to mind as something more than a natural phenomenon is illegitimate (Urmson and Ree, 1989, p. 218).

This definition makes the ontological implication that there is only what we call “nature” and what the sciences study. But what is it that we denote by the word “nature”? The definition does not make any positive statement as to its character. Thus, the definition is compatible with the existence of deities, values, or minds, *if* these are considered as natural (or part of what we call “natural”). For example, a pantheistic theory where God is considered as identical with nature is compatible with naturalism thusly defined (*cf.* Spinoza’s pantheism; see also Sjöstedt-H, 2022). However, commonly naturalism is taken to imply physicalism, and it is unclear whether, or in what sense, entities like deities could belong to the extension of the term “physical.” This is a question that we will turn to in due course.

The *Cambridge Dictionary of Philosophy* (edited by Robert Audi), distinguishes between the methodological and metaphysical component of naturalism:

Naturalism, the twofold view that (1) everything is composed of natural entities – those studied in the sciences (or in some versions the natural sciences) – whose properties determine all the properties of things, persons included … and (2) acceptable methods of justification and explanation are commensurable, in some sense, with those in science. Component (1) is metaphysical or ontological, component (2) methodological and/or epistemological (Audi, 1995, p. 517–518).

Like the first definition, this one defines the ontological nature of the natural as *that something* – whatever it is – that the sciences study. The methodological component is, by contrast, weakly defined as “commensurable” with the methods of science. This is a very liberal definition and could mean every method that is *not incompatible* with the findings and methods of mature science. This can be taken to entail both that our *methods* of knowing nature should be compatible with science, and that the *theses* or *theories* that we form based on these methods should be consistent with mature scientific theories.

The ontological part of naturalism does not rule out that there could be facts about nature that are not strictly scientific^7^, as long as our formulation of these facts does not contradict physicalism or the contents of mature scientific theories. Using the terminology of Orr (2006), we can say that there could be facts that are *non*scientific (i.e., outside the scope of science), but not *un*scientific (i.e., incompatible with science). Such facts could pertain to God, morality or consciousness, on the premise that science alone does not entail whether God exists or not (and in what sense), what is right or wrong (*cf.* Hume’s guillotine which states that it is not valid to infer from how things are to how things ought be), or what it is like to be subjectively conscious (Chalmers, 1995). Such facts would be outside the domain of what can be studied in science. They need not conflict with the ontological part of naturalism, unless we consider God, morality, or consciousness to be something nonphysical or supernatural. There are viable philosophical arguments to the conclusion that such facts can be considered as natural [*cf.* (Rosen, 2017) regarding moral naturalism, or (Strawson, 2003, 2006) concerning naturalism about consciousness, or (Stone, 2012) on spiritual naturalism]. In contrast, the epistemological aspect is less clear: is our way of *knowing* such nonscientific facts compatible with science? This depends on how the epistemological access to such facts is elaborated. As we will see, the epistemological problem becomes central when we turn to mysticism. In any case, it can be argued that it is at least *possible* that there is knowledge that is extra-scientific.

It would be a logical fallacy to infer that if something (e.g., the existence of God, morality, or consciousness) does not follow from science, then it cannot exist. Such a conclusion does not follow from naturalism, the scientific method, or scientific theories, without the extra premise that there are no facts beyond those described by science. This premise is closely related to scientism, that is, the epistemological thesis that science, usually equated with natural science, is the best or only way to attain truths about reality.

Scientism can be divided into *weak scientism*, which holds that science is the best way to know reality, and *strong scientism*, holding that science is the *only* way to know reality. Further, scientism can be divided into *broad scientism* where science is considered to include fields such as the social sciences and humanities, and to and *narrow scientism* where science is equated with the natural sciences (Hietanen et al., 2020). From the perspective of naturalism, it is plausible to focus on narrow forms of scientism where epistemological priority is given to the natural sciences, on the assumption that all that exists is grounded in fundamental physical processes. Regarding weak and strong versions of scientism, strong scientism categorically denies the possibility of extra-scientific knowledge, whereas the weak version merely prioritizes science. Both the weak and strong theses can be considered to contradict our knowledge of, for example, moral facts, given that what is right or wrong cannot be known through natural science *at all* (unless one endorses naturalistic reductivism about moral facts and our knowledge of them; see Lutz and Lenman, 2018). Science can merely answer what people *consider* as right or wrong, but such observable facts are different from what really *is* right or wrong (if anything) which, in turn, is a philosophical question. Similar reasoning could be applied to aesthetics or religion, but that discussion is beyond the scope of this paper.

**Figure 1:**
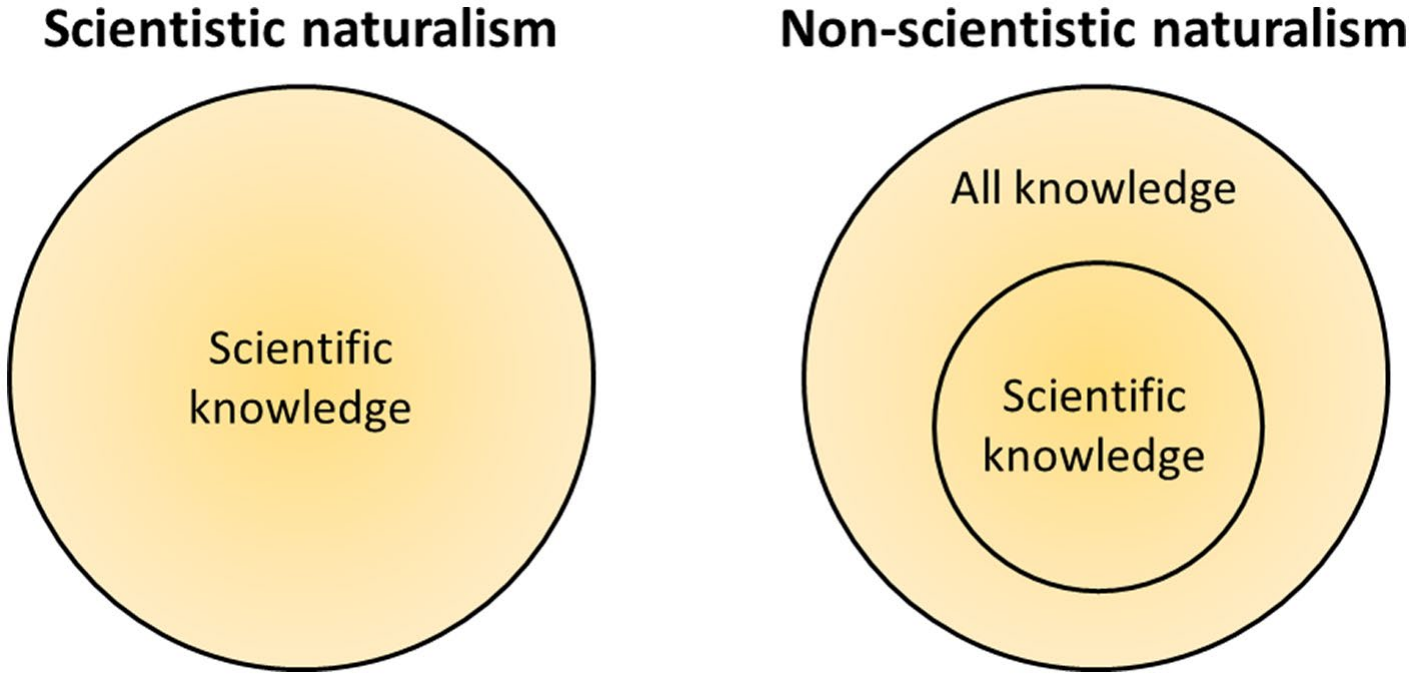
Scientistic naturalism holds that science can capture all there is to know about nature. Non-scientistic naturalism implies that there can be more facts of nature than what science can convey, as well as, potentially, more knowledge of nature than just scientific knowledge. (Note that there could also be facts that are not knowable at all, in which case no type of knowledge could capture all facts of reality.)

To illustrate the limits of scientism, we may focus on the case of consciousness. It seems plausible that I know my own consciousness – to take a trivial example, what coffee tastes like – without any resort to science, natural or humanistic. It can be argued that science is not the only way nor even the best way to know what coffee tastes like, given that from science alone we cannot infer that subjective, phenomenal consciousness exists at all (Nagel, 1974; Jackson, 1986; Chalmers, 1995). This yields a contradiction even with weak and broad forms of scientism, since not even humanistic or social sciences could convey the taste of coffee to someone who has never tasted it. And still, the taster knows the taste, even if they had never known of any form of science. In short, the case of consciousness demonstrates that there can be more knowledge and facts than what is implied by science (Figure 1).

## 4 Consciousness and the limits of science

Non-scientistic naturalism implies that there can be facts of nature that are not captured by science alone, but which can be known in other ways; that is, there can be extra-scientific knowledge. The domain of facts is broader than the domain of scientific facts, and the domain of knowledge is broader than the domain of scientific knowledge (Figure 1). If we assume, following the ontological component of naturalism, that everything is physical, then the extra-scientific facts would nevertheless be *physical* facts. This implies that there is more to the physical than what physics, or science generally, can capture. This is a central thesis in Galen Strawson’s version of physicalism (Strawson, 2003, 2006). In his view, consciousness is a real and concrete phenomenon, which is physical on the premise that all concrete and real entities are physical. Strawson calls this thesis *realistic physicalism*, that is, physicalism that is realistic about consciousness, and contrasts it with *physicSalism*, the thesis (or *faith*, as he calls it) that physics and (natural) science can capture all aspects of the physical. He argues that physicSalism is undermined by the case of subjective consciousness, whose nature science cannot fully capture. This is known as the explanatory gap in philosophy of mind (Levine, 1983), also known as the epistemic gap. Here I use the term “epistemic,” as it can be considered as broader, on the assumption that there is more knowledge than explanatory or conceptual knowledge.

Innumerous arguments throughout history of philosophy demonstrate the epistemic gap. For example, Bertrand Russell wrote as follows:

It is obvious that a man who can see knows things which a blind man cannot know; but a blind man can know the whole of physics. Thus the knowledge which other men have and he has not is not part of physics (Russell, 1927, p. 389).

I assume that the reader is familiar with other, more detailed arguments for this conclusion, and it suffices to mention them here without going into the details. For example, Thomas Nagel argued influentially that even if we knew every scientific fact about bats, we could nevertheless not know what it is like to *be* a bat (Nagel, 1974). Thus, there is a massive range of facts of subjective phenomenal consciousness that science cannot capture. Likewise, Frank Jackson made an argument regarding the neuroscientist Mary, captured in a black-and-white room (Jackson, 1986). To convey Jackson’s point without facing some problems specific to that thought experiment (e.g., Mary could see her own blood), we may imagine a completely color-blind neuroscientist Mary who knows all there is to scientifically know about color perception: the visual system in the brain, neural correlates of consciousness, neurophysiology, and even physics in general. Arguably, if she were to somehow acquire color vision (say, in a surgical operation), it seems evident that when she for the first time *sees colors*, she learns something new: what colors look like. If she learns something new, then that something was not captured by her previous scientific knowledge. By the same token, a psychedelically naïve scientist, knowing all scientific facts of psychedelic experiences in the brain, nevertheless learns something new when she personally undergoes a psychedelic experience (Jylkkä, 2022). Finally, Chalmers (1996) argues that it is possible to conceive of physical duplicates of actual humans or “zombies” which lack phenomenal consciousness, such that there is nothing it is like to be a zombie. Chalmers takes this to show that phenomenal facts are not determined by physical facts alone.^8^

Chalmers and Jackson take their arguments to show that consciousness cannot be physical, but this conclusion is widely disputed. However, a less controversial claim is that all the above thought experiments demonstrate the *epistemological point* that subjective consciousness cannot be known through science alone. This is a widely accepted notion. The only position in philosophy of mind that denies that we have knowledge of what experiences feel like is eliminativism or illusionism, which deny the existence of subjective, phenomenal consciousness altogether (Dennett, 1991; Frankish, 2017). Discussing that position is beyond the scope of this paper, and I will assume it as a trivial fact that we do know what experiences feel like: they feel like *this*. *This* is what it feels like to philosophize and listen to John Coltrane. But what is *this*, and what does it tell us about the nature of reality?

### 4.1 Physicalism with a Kantian twist

Suppose that consciousness is indeed physical as the naturalist claims. Consciousness can be considered as a concrete and real physical phenomenon – indeed, of all the physical phenomena, for us it is the most real and its existence is beyond doubt. There is extensive empirical evidence for this position from neuroscience: conscious experience can be altered through manipulating the brain, and there is no evidence that changes in consciousness could take place without corresponding changes in the brain (or some other physical substratum^9^). It is plausible that eventually neuroscientists will discover the *constitutive mechanisms of consciousness* (CMC) such that they directly correspond to experiences, the two being perfectly structurally isomorphic (Revonsuo, 2006; Jylkkä and Railo, 2019). The integrated information theory can be considered as a step into this direction, arguing that every experience is perfectly isomorphic to an information structure, described as a maximally integrated conceptual structure, which in turn corresponds to a process in a physical system such as the brain (Tononi et al., 2016). It must be granted that we do not yet know how consciousness should be described in (neuro)scientific terms but based on the current evidence it is highly plausible that consciousness is physical. After all, if consciousness was not physical, it could not interact with the physical reality due to the causal closure of the physical (and I assume that consciousness does have causal properties, e.g., my thirst causes me to seek something to drink). It can also be argued *a priori* that consciousness must be physical on the premise that all concrete and real phenomena in the world are physical (Strawson, 2006).

The naturalistic thesis that consciousness is physical is more radical than commonly perceived. In fact, it is nothing short of *miraculous*. How could subjective consciousness – the taste of coffee right now and these thoughts flowing in my consciousness – be *physical*, that is, forms of the same substance as the stars, this planet, and the coffee itself? This claim is made even more miraculous by the fact that science alone cannot convey to any other person how coffee tastes like for me, or what it is like to entertain these thoughts. Consciousness demonstrates that the physical is more than what the sciences can convey. In the light of the epistemic gap and on the premise of physicalism, it can be argued as follows:

P1. Consciousness is physical

P2. We know what consciousness feels like

P3. Science cannot convey what consciousness feels like

C. Thus, there are facts about the physical that cannot be captured by science alone.

In other words, science is epistemically limited: there is at least one kind of knowledge that is outside its scope, or that is nonscientific. This leads to the question of what kind of physical phenomenon subjective consciousness is, and why science cannot capture it. Answering this question leads to vast metaphysical landscapes where the paths are many and one easily gets lost. However, even if there is no definite answer to *why* science cannot capture the nature of consciousness, we may start from the premise that this epistemic limit of science is *real*: there are physical facts that science cannot convey, even if we do not know exactly why that is. Next, my aim is to briefly illustrate some answers to this problem, although I cannot go into them in much detail. I take it that the case of consciousness reveals the limits of science and that nature is more than what science alone implies. The relevance for the case of mysticism is this: reality can, in some sense, be taken to have a “fundamental nature” that is beyond the scope of science, and this nature includes consciousness (*cf.* the easy problem, EP1). Moreover, we have direct access to this part of the fundamental nature of reality through being conscious (*cf.* the hard problem, HP). Now the task is to elaborate what this means.

### 4.2 Russellian monism

Russellian Monism (RM) is one way to elaborate how consciousness is beyond the scope of science. It is noteworthy that RM is often contrasted with physicalism (e.g., Goff, 2017) but it can also be given a physicalistic interpretation, as Strawson does (Strawson, 2003, 2006, see also Montero, 2015). According to RM, science is limited to modeling *extrinsic properties* which are generally considered as relational or structural. For example, the Newtonian equation *F = ma* specifies the interrelations between the variables of force (*F*), mass (*m*) and acceleration (*a*), or Einstein’s equation *E = mc^2^* defines how energy (*E*) and mass are related, and so on. However, none of these equations can tell, according to RM, what entities such as force or energy are intrinsically, that is, beyond their relations to other entities. It is intuitive to suppose that an entity like an electron has some nature or essence in and of itself, which is the way it is considered apart from its relations to other entities. For example, we can observe how electrons behave in electromagnetic fields – how their acceleration and direction change, as happens in old-fashioned CRT-televisions – but it seems intuitive that electrons have some nature independently of how they are disposed to behave in electromagnetic fields, or independently of how they are disposed to interact with *anything*. RM holds that such relational or dispositional properties of an entity are grounded in the categorical or intrinsic nature of the entity. The logic is that if objects A and B are disposed to interact in a specific way, this must be grounded in the way A and B are in themselves, independently of the relation they have to each other. Thus, scientific observation and modeling can only capture the extrinsic properties of matter, but not their intrinsic bases. As Goff has crystallized the point, science can only tell us what matter *does*, not what it *is*. In sum, RM holds that consciousness is an intrinsic property and therefore beyond the scope of science.

If the intrinsic properties postulated by RM are physical, then RM can be considered as compatible with naturalism. It could, however, be argued that RM is in some respects incompatible with the *spirit* of naturalism. It postulates the existence of two distinct types of properties, roughly mental (intrinsic) and non-mental (extrinsic), leading to a kind of property dualism (Kind, 2015; Chalmers, 2019). This leads to several dualistic problems, such as how do the intrinsics have any causal power or how “mental causation” is possible (Howell, 2015), or how the intrinsic and extrinsic properties are related (Hiddleston, 2019). In my view, the most serious problem of RM is that it is ontologically heavy: based on *a priori* reasoning, it essentially doubles the number of properties in the world, postulating an intrinsic categorical property in addition to each extrinsic property, whereas for scientific explanation the extrinsic properties would suffice. RM ties us to a very specific kind of substance ontology with static intrinsic properties and is incompatible with process metaphysics where happenings and interactions are prior to objects and properties (Jylkkä and Railo, 2019). It could be argued that modern quantum mechanics, claiming that the fundamental nature of reality is best described as a wave function, is best compatible with, or even leads to, a process ontology, where there are no definite particles or things which could have purely “internal” properties (Rovelli, 2021). Indeed, many naturalistically-minded philosophers reject the notion of intrinsic properties and endorse ontic structural realism instead, where the relations described by science are considered as ontologically fundamental (Ladyman, 2023). An extensive discussion of the problems of RM and its compatibility with modern physics and naturalism is beyond the scope of this paper. However, it can be argued that RM is based on a more general, Kantian idea about the limits of observation, which need not be coupled with categoricalism or the existence of intrinsic properties.

### 4.3 Naturalistic monism

According to Naturalistic Monism (NM), consciousness is identical with its neural correlate or constitutive mechanism, and the epistemic gap between the two reflects the distinctness between the scientific model (e.g., the “neural correlate”) and the modeled itself (i.e., consciousness) (Jylkkä and Railo, 2019). The epistemic gap is thus not specific to consciousness but reflects a *general* limit of science: its limitedness to modeling reality based on observations. This Kantian intuition is vividly captured by the 19^th^ century astrophysicist Arthur S. Eddington, who writes as follows:

The atom is, like everything else in physics, a schedule of pointer readings. The schedule is, we agree, attached to some unknown background. Why not then attach it to something of a spiritual [i.e., mental] nature of which a prominent characteristic is thought. It seems rather silly to prefer to attach it to something of a so-called ‘concrete’ nature inconsistent with thought, and then to wonder where the thought comes from. We have dismissed all preconceptions as to the background of our pointer readings, and for the most part we can discover nothing as to its nature. But in one case – namely, for the pointer readings of my own brain – I have an insight which is not limited to the evidence of the pointer readings. That insight shows that they are attached to a background of consciousness. I may expect that the background of other pointer readings in physics is of a nature continuous with that revealed to me in this way (Eddington, 1929, pp. 258–260).

Importantly, Eddington does not postulate the existence of any separate class of intrinsic properties that consciousness is part of, but rather emphasizes how science is limited to *modeling based on observations*. According to NM, there do not exist ontologically distinct intrinsic and extrinsic properties, but rather there is only the *epistemic* difference between how things appear to us in observation and thought, versus the way things are independently of being observed and modeled. Like all human beings, scientists cannot but represent the external world in their minds; they cannot step outside their consciousness to see how things are independently of their own consciousness. Thus, NM is compatible even with ontic structural realism or process ontology where no intrinsic properties are implied.

The main idea underlying NM can be illustrated with the classical example of a tree falling in forest when there is no one there to hear it. Does it make a sound? The naturalist might say that it does make a sound, in the sense of there being specific kind of vibration of air molecules, but there is no sound as sensation or brain process. However, what is the vibration of molecules when there is no one to model it as “vibration of molecules”? The scientific account of sound is merely a representation in our minds, and our “vibration of molecules” -theory is something distinct from what it is about. The vibration of molecules as a process independent of the human mind is the *object*^10^ of the scientific representation, distinct from the representation. What is the representation-independent nature of that object? That is, what is the nature of the object independently of being conceived of as “vibration of molecules”? This is a mystery; it is *logically impossible* to know the answer through representation. The moment I try to understand the mind-independent nature of an object, I bring it to my consciousness. In short, the scientific model is distinct from the modeled and does not afford us access to its nature in itself, independently of being modeled and observed in a specific way. Our ignorance of things in themselves can be called “Kantian humility” (Langton, 2001).

Kantian humility can be elaborated in several ways. For example, the Kant scholar Lucy Allais writes that “the way the object is presented in consciousness is *something more* than the object alone, as it is outside of this relation” (Allais, 2015, p. 113, emphasis added). On this approach, perception of an object is constituted by the interaction between the perceiver and the object. That is, when a scientist observes, say, an atom, the way the atom appears to them is constituted by the way they and their measuring devices interact with the atom itself, that is, that something “out there” that exists independently of being observed (although not necessarily independent of how it is related to *other* entities besides the observer^11^). This idea is illustrated by the measurement problem in quantum mechanics (QM). In QM, an elementary particle is modeled by a wave-function whose mechanics (e.g., spatial position, temporal development of different properties) are described by the Schrödinger equation. The Schrödinger equation merely specifies the probability of making certain *observations* but cannot tell anything of what underlies the observations, when it is not observed. There is no way of knowing what the wave function is “in itself,” that is, beyond our observations and models of it. The act of measurement can be considered to render the observed and the observer parts of a new composite system, constituted by the “observer” and the “observed” causally interacting with each other.

Another way to illustrate the notion that we cannot know what underlies our observations and models, even in science, can be framed in terms of the predictive coding theory in neuroscience (Friston, 2010; Hohwy, 2013). This account implies that our consciousness is a model of reality, generated by the brain to predict observations and thereby to increase the organism’s fitness. This happens in a Bayesian inference process where the brain aims to minimize surprise or free energy (roughly equivalent to informational entropy) through making best guesses about the *hidden causes* of observations. The hidden causes are the external objects that affect our senses, but which we have no direct access to. This account appears to corroborate the Kantian notion of our ignorance of things-in-themselves, even when it comes to science (Swanson, 2016). If our scientific conceptions of atoms and quarks are brain-generated models, how can we know whether they correctly or exhaustively describe reality? As philosopher Dan Zahavi writes:

It is not altogether clear how [a scientific realist] so confidently can declare that whereas the world of experience is a brain-generated illusion, the world as described by physics, the world of electromagnetic radiation, is the world as it truly is (Zahavi, 2018, p. 53).

Zahavi then examines several possible arguments that the scientific (naïve) realist might make to the conclusion that the world really is as science describes it and argues that these do not hold. However, independently of what one thinks about the correctness of scientific models, or their correspondence with reality, a more fundamental point remains: we should acknowledge that even *correct* models of reality are *still just models*, and reality outside the models remains a mystery. Magritte’s pipe in *The Treachery of Images* is a correct picture of a pipe, but it still is not a pipe, and even a correct neuroscientific model of pain is not pain. What predictive coding demonstrates is that *all* our conceptions of reality, scientific theories included, are brain-generated models similar to dreams or hallucinations, a map distinct from the territory. The territory in itself, the nature of reality beyond our models of it cannot be known through representation.

What is nature, then? What is it that our words “nature” and “physical” refer to? As Strawson emphasizes, the word “physical” is the “ultimate natural kind term” (Strawson, 2019). According to the causal-historical theory of reference, currently the dominant position in philosophy of language, a natural kind term *t* refers to *that something* that is instantiated by the actual samples that we refer to as *t*, or to which we are causally linked in the act of naming (Kripke, 1980). Thus, “physical” refers to *that something* instantiated by atoms, electrons, water, neural processes, and so on. Importantly, the causal-historical theory of reference implies that a natural kind term refers to that something, and nothing but that something, even if we did not know its fundamental nature. For example, it could turn out that we have dramatically erred about the nature of water, so that it is not after all H_2_O, but rather some exotic, unknown compound XYZ (Putnam, 1975). Even in this case, the term “water” would refer to XYZ and nothing but XYZ, despite our erroneous conception of it as H_2_O (on Kripke’s terminology, natural kind terms are *rigid designators* and not dependent on our conceptions of their referents). Again, even if our conception of water as H_2_O were correct, the causal-historical account implies that its fundamental nature or “essence” can be *more* than what science conveys. Indeed, it can be argued that the term “neural correlate of consciousness” is a natural kind term, and that the fundamental nature of what it refers to is, at least partially, constituted by consciousness.^12^

**Figure 2:**
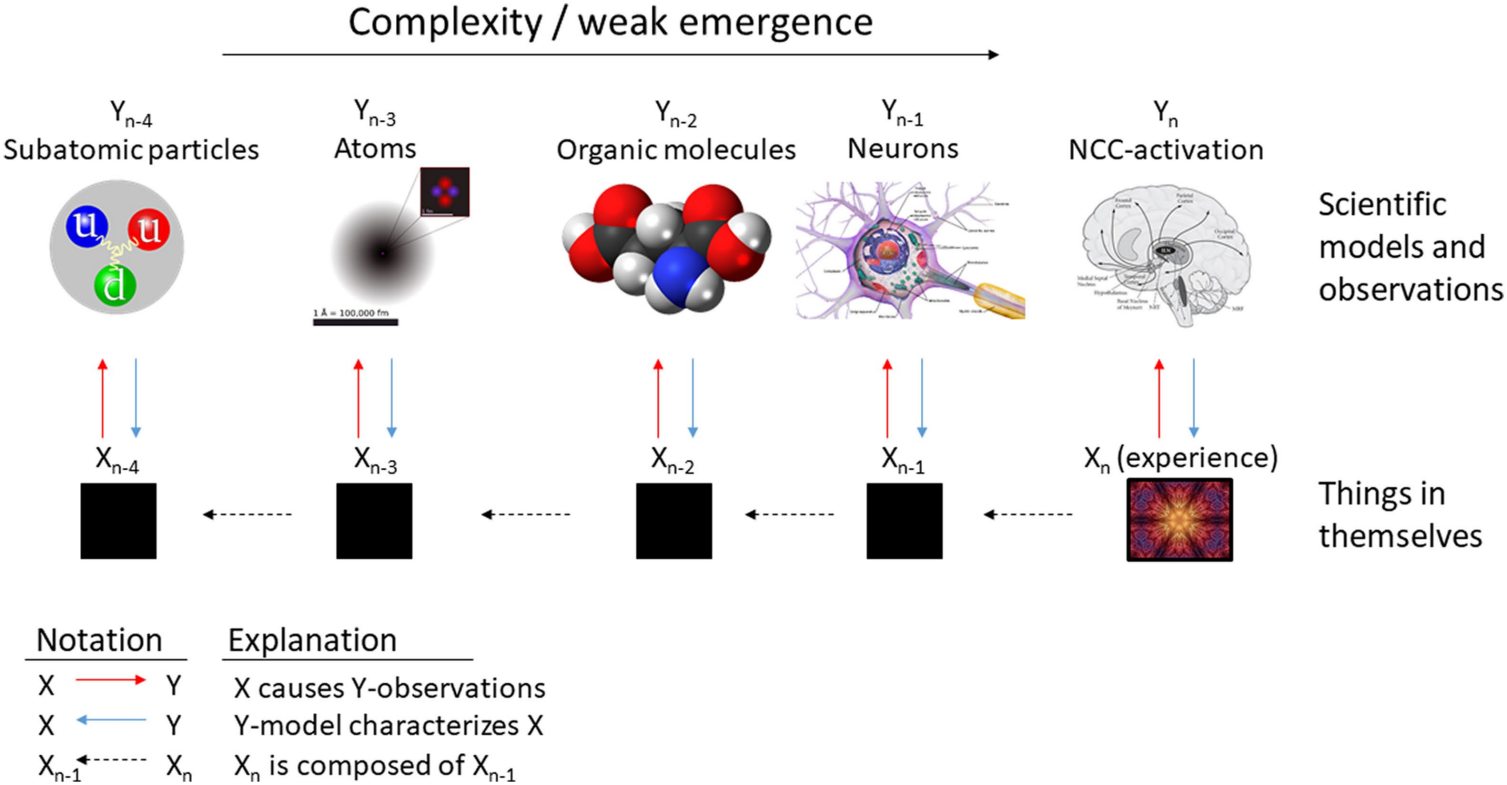
Consciousness, depicted here on bottom right as a specific type of experience (*X*_n_), is identical with its neural correlate (NCC on level *Y*_n_) in the sense that the NCC-model represents the experience type. Neuroscientific observations of NCCs are *caused by* the experience *X*_n_ and the NCC-models are *about* the experience. However, the scientific observations and models do not yield direct access to the hidden causes of the observations, which in the case of the NCC is the conscious experience. More generally, consciousness (*this*) is the “thing-in-itself” that underlies neuroscientific observations of NCCs. Consciousness can be depicted as a macroscopic process (*Y*_n_) that is based on, or can be reduced to, lower-level processes (*Y*_n-x_). These models (Y) are representations of the things in themselves (X). I only have direct access (at least normally) to the single physical process that is my consciousness, hence the black boxes. However, assuming that strong emergence is impossible, there is a continuum between consciousness (*X*_n_) and its constituents (*X*_n-x_), implying that the constituents of consciousness, including the ultimate physical entities, are of the same general kind as consciousness. Adapted from Jylkkä and Railo (2019).

To illustrate, suppose that you were a subject in a neuroscientific experiment on the neural correlates of color vision. You enter the fMRI device and your task is to imagine different colors. When you imagine red, activation pattern X emerges on the neuroscientist’s computer screen. When you imagine blue, another activation pattern Y emerges, and so on. What is the “hidden cause” of the neuroscientist’s observations? The neuroscientist does not know, but you do: it is your imaging of red, blue, and so forth. It is your *experience* that produces the image of the activation pattern. You can voluntarily produce different images of neural activation at will, a vivid example of mental causation.^13^ This can be taken to suggest that the hidden cause of the fMRI-data *is* your consciousness. Indeed, this is what a materialist identity theory would say: if consciousness is *identical* with its neural correlate, then what we refer to as “neural correlate” from the outside *is* consciousness, when considered from the internal or subjective perspective. What makes the two, the objective and the subjective perspective, appear so different is that the former is a *model* of the latter. It is subjective consciousness that neuroscientific models of its “neural correlates” are *about*; it is subjective consciousness that *produces* scientific observations of its physiological mechanisms (Jylkkä and Railo, 2019).^14^ Thus, in the case of consciousness we can have direct access to what underlies the scientific pointer readings, unlike – or so it appears – in the case of any other processes in the universe (Figure 2).

In sum, it can be argued that through representing reality, we are left in ignorance about the representation-independent nature of reality, or what reality is in itself. This representation-transcendent nature of reality can be considered as its “fundamental nature,” as referred to in the Core: it is beyond all observation and representation. This presents a tentative answer to the easy problem of mysticism (EP1). Moreover, we can know this fundamental nature of reality directly, at least in the case of our own experience: it is *this* process here and now, happening as I write these words. I know my consciousness through *being* it, not merely through introspectively representing it. This reasoning may pave the way to answering the hard problem (HP), although the problem is still to show how one’s own experience could reveal the nature of reality at large, beyond one’s own consciousness or brain.

### 4.4 Unitary vs. relational knowledge

Scientific knowledge, as all representational knowledge, can be considered as *relational* in that the representation is *about* its object, the two being separate (Jylkkä, 2022). For example, my experience that there is a cup on the table is *about the cup itself*, the hidden cause of my observation, to which I have no direct access to, and which I merely represent in my consciousness, or know through interaction with it.^15^ As to all entities in the world besides my own consciousness, my access is relational and limited to the “pointer readings,” that is, models and observations. What underlies them, considered independently from the human process of observation and modeling, is a mystery; hence the black boxes in Figure 2. However, it can be argued that I can know my own consciousness directly through *being* it, in a unitary way.^16^ I am not *related* to my consciousness, I *am* my consciousness. Consciousness is *this*, the sound of Mozart’s piano sonata, the sensation of the body, the taste of coffee. Although these experiences are expressed in terms of a subject having the experience (“I” hear the music, “I” sense my body, “I” taste the coffee), the division between subject and object can be considered as artificial. There is merely the experience of music happening in the here and now, it is *this*. Both my conception of myself as a subject, and the music as a distinct “object” are all experiences in a single, unified consciousness, *this*. All my experiences are *in this consciousness* (i.e., parts or modifications of *this*) and thus they cannot be the *objects* of consciousness, when by “consciousness” we mean consciousness in its totality (i.e., what happens here and now, *this*). Notice that this does not preclude the possibility of reflectively or relationally knowing our experiences. In reflection, it can be considered that one part of consciousness (e.g., a higher-order thought) takes another part (lower-order sensation) as its object, as in reflecting on the taste of the coffee. However, in addition to reflectively knowing the taste, we can also know the taste simply through tasting, non-reflectively. Note that we non-reflectively know even the process of reflection taking place. One arguably knows that they are reflecting without the need to reflect on their act of reflection; denying this would lead to infinite regress where one would never know that they are reflecting. I know that reflection is *this* process here. When we attend to consciousness in its totality, we see that subject and object, the reflection and the reflected, are all in one consciousness (*this*), which we know unitarily through *being* it. There is simply the flow of consciousness in the here and now.

Consciousness in its totality is always unitary, however I often do not notice this unity because I am focused on what I perceive as individual objects, distinct from myself. There is the coffee which I can sip and the keyboard which I write on. However, I can also choose to focus on my consciousness as a totality, *this*, and see that this is unitary. When I look at my conception of myself, I notice that it is nothing but an experience in consciousness; when I look at my perception of the cup, I see that it is just a sensation. They are all parts of *this*. From the cognitive perspective it could be said that the self is a generative model produced by the brain to enable functioning of the organism in the environment (Letheby and Gerrans, 2017). When the organism perceives “self” as distinct from “external” objects, it can hunt some objects and reproduce with others, and this could not take place without perceiving subject and object as distinct. However, experiences of the self and other, subject and object, are nothing but brain-generated models, and can be argued to be accidental, not essential to consciousness. Consciousness can also take place without such models, as demonstrated by psychedelic experiences where the models can be considered to dissolve (Carhart-Harris and Friston, 2019). However, it is not necessary for the self-model to dissolve in order to see the unitary character of *this*. In meditation, be it psychedelically assisted or not, we can attend to the totality of what we experience, seeing that it is an undivided whole (and paradoxically, then there is no “I” that sees the totality, but just the totality, *this*). This does not amount to an “ego death,” at least not in the radical sense of collapse of the self-model, which is often experienced as deeply frightening. Even when the subject and object do not radically dissolve into each other, one can see that they are both interconnected parts of the same unitary consciousness, *this*.

I have argued previously that this type of unitary knowledge is demonstrated most vividly by cases of unitary consciousness in psychedelic and mystical experiences, where the commonly experienced subject-object division vanishes due to the collapse of the self-model (Jylkkä, 2022). This point is also emphasized by Forman (1993), who uses the concept *knowledge by identity* to refer to how one can know a pure conscious event that lacks all content. However, I take it that unitary knowledge is present in *all* experience, although it is most vivid in unitary experiences (be they internal or external, to follow Stace’s terminology). For example, Michael Pollan describes his psilocybin experience as follows:

[I] lost whatever ability . . . to distinguish subject from object, tell apart what remained of me and what was Bach’s music . . . I became a transparent ear, indistinguishable from the stream of sound that flooded my consciousness until there was nothing else in it (Pollan, 2018, p. 254).^17^

Although in this example there is no “subject” that knows the “object,” no “Michael Pollan” who experiences the music of Bach, there appears to nevertheless be *knowledge*. Something *happened* in what we refer to as “Michael Pollan” during a specific temporal duration, and this *happening* was knowledge. Pollan could have referred to this knowledge-constituting process as “this” at the moment it took place to somehow ostensively capture its unitary nature (a gesture similar to the facial expression of awe), but in order to know the experience, Pollan need not consider it as “this” or as *anything* (it could be said that the experience is *no-thing*). It is difficult to characterize this process conceptually because the syntax of language operates with subjects, predicates, and objects. In unitary knowledge or *this*, one knows without knowing anything, and without there being anyone who knows. Unitary knowledge is not *about* anything; it is simply the happening of an experience in the here and now; it is pure awareness.^18^ Thus, it is different from what Russell called knowledge by acquaintance, which he defines as the most direct *relation* a subject can have to an object (Russell, 1910, p. 108). Although experiences of ego dissolution most clearly demonstrate the notion of unitary knowledge, it is plausible that we *always* know consciousness in this unitary way; as some Zen scholars put it, the everyday mind is already the “enlightened” mind. *This* is knowledge of what experiences feel like. *This* is knowledge that requires no concepts, *this* is not *about* anything (i.e., *this* is non-intentional), *this* is non-reflective.^19^

It is worth noting that unitary knowledge is quite different from knowledge as traditionally conceived. It could be objected that due to its radical differences with conceptual or representational knowledge, it does not deserve to be called “knowledge” at all. However, I take it that unitary knowledge is the most fundamental type of knowledge: it is simply consciousness and can be argued to be a prerequisite for all other types of knowledge (see Forman, 1993). Another possible objection to unitary knowledge is that it is “encapsulated” and private, and therefore cannot inform (conceptual) representations. However, this objection would be based on a misunderstanding of what unitary knowledge is. It is simply consciousness in the here and now, which from the naturalistic perspective can be considered as a causal process in the brain. Thus, it can be causally linked to other processes in the brain, such as the cognitive-representational system. An experience E (i.e., a piece of unitary knowledge) can be the object of a representation R, and while R necessarily fails to capture what E is independently of being represented as R, there can nevertheless exist a causal link between E and R. For example, I can remember my experience of cycling to work, which I unitarily knew as I was cycling, but which now is merely the object of my present cognition.

## 5 Mystical-type insight and naturalism

I have argued that there is a sense in which reality can be said to have an ultimate nature: it is the nature of reality in itself, beyond our representations and models of it. Thus, it is is non-accessible by science, which is limited to representing objects. This ultimate nature can be considered as unitary in the sense of substance monism: everything that exists are forms of a single type of substance or process, or what we model as the “physical.” Science merely represents reality based on “pointer readings” and does not afford us insight into the nature of reality as it is beyond our representations and observations. This idea of Kantian humility can be elaborated in line with naturalism, and one possible way to do that is in terms of Naturalistic Monism. Moreover, I have argued that we can know our own consciousness in a direct, non-representational, non-conceptual, non-intellectual, and unitary way simply through being it: it is *this*, what happens in the here and now every moment.

We have arrived at an important semi-conclusion: *This* can be considered to reveal the ultimate (i.e., representation-independent) nature of reality, but only for the part that is my consciousness, or its neural correlate. In other words, *this* already amounts to mystical-type knowledge where we know the fundamental nature of reality directly, through being one with it – albeit only for the part of reality that is my consciousness (or specific types of neural processes). And by *this* I do not mean any exceptional experience, but simply *experience* in the here and now: this process of writing and reading, thinking, sitting, hearing the music. Now the question is, what is the role of psychedelics in all this? It can be argued that psychedelics facilitate seeing the nature of consciousness in itself, or *this*. Although *this* is present every moment, we often do not notice it, and psychedelics can facilitate becoming aware of *this*, thus facilitating mystical-type knowledge of the fundamental (i.e., representation-independent) nature of reality.

Mystical-type experience and psychedelics may facilitate acknowledging the limits of our models of reality, and to become aware of the intrinsic nature of consciousness. On the neurocognitive level, psychedelics arguably loosen high-level beliefs or “priors,” enabling increased bottom-up information flow (Carhart-Harris and Friston, 2019). The loosening of priors could even lead to what can be called *model collapse*, or the bypassing of habitual patterns of thought and perception. This can involve the collapse of our models of consciousness and reality. Psychedelics could thus enable us to better see the ineffable nature of consciousness as it is in itself (*this*), that is, to become aware of the nature of consciousness beyond our reflective models of it. This is most vividly illustrated in unitary experiences, where the subject-object division collapses altogether. Note, however, how language leads us astray here. Strictly speaking, in in a unitary experience there is no “us” who become aware “of” consciousness as it is in itself; rather, there happens what could be conceptualized as the *becoming of this*: entering a state of consciousness where the habitual models do not apply, and only ineffable consciousness remains. Another way to formulate the process would be to say that when there is no longer any model M such that consciousness could be conceived of *as* M, then what remains is simply consciousness in itself as a totality, which we know through *being* it rather than through modeling it. It remains unanswered how exactly everyday consciousness differs from *this* (consciousness in itself), and following some Zen scholars, it could be argued that there is no difference: we cannot help but *being* consciousness in itself every moment, because consciousness in itself simply is the totality of one’s experiences. Nevertheless, there seems to exist a difference between *this* and our innumerous representations *of* this – and notice that, strictly speaking, even the representations are part of *this*. How to formulate *this* remains an open question, and answering it is made difficult by the fact that language is necessarily dualistic: there is the word and the referent, and the syntactic structure that differentiates between subject, predicate, and object. However, in direct experience these limitations can arguably be transcended, which enables the becoming of *this*. Psychedelics can facilitate attaining this unitary state of consciousness, or it could also be said that psychedelics “amplify” *this* (Jylkkä, 2022).

It could be argued that the “becoming of this” takes place through becoming aware of the limits of our models. Through model collapse, psychedelic or mystical-type experiences can show the limits of words and representations and show how our models of reality are a map, distinct from the territory. Psychedelic or mystical-type experience can lead us to become aware that our notions of, for example, reality as “physical” or mind as “neural process” or “predictive coding” are all just concepts in our minds, leading to the question: what are the entities referred to by these terms in themselves? It could be said that psychedelics lead to a global *cognitive defusion*,^20^ where we see our models of reality for what they are: a map that is distinct from the territory. Another way to formulate this point is that typically our models of reality are *transparent* and act like a lens through which we see reality. In psychedelic experience, by contrast, the models may become more *opaque*, enabling us to become aware of them *as* models. However, being conscious *of* models *as* models is still a dualistic mode of consciousness where we are limited to representing, but it can be a crucial step in the becoming of *this*. Similar to a Zen koan, seeing models *as* models can lead to seeing *all* modeling as futile. Through clearly seeing how mind cannot attain itself, and through realizing that trying to attain the mind by using the mind leads to infinite regress, we can come to notice that there is no point in trying to grasp the nature of the mind. There is no point in attempts to understand *this* because *this* has been present all along. We have always directly known *this* part of reality since we are it.^21^

In sum, psychedelics may facilitate us to see our models *as* models and to acknowledge their limits. This can be considered as a *negative epistemic benefit*: we do not know the nature of reality beyond our models, it is a mystery. Moreover, psychedelics may facilitate the *positive epistemic benefit* through enabling us to become aware of *this*: to know consciousness in a unitary and direct way without modeling it. This can be taken to afford us direct access to the ultimate nature of reality insofar as that reality takes the form of human consciousness.

### 5.1 Tackling the hard problem

Through model collapse and cognitive defusion, psychedelic experience (or mystical experience generally) can lead us to ask: what *is* the physical? Then we can come to realize that *this* is the physical, or at least one instance or modification of it. Even if this enables a glimpse only to one specific form of the physical as it is in itself, this already entails a major epistemic achievement, as it enables us to unitarily know at least a part of the fundamental nature of reality (i.e., the nature of reality beyond models and conceptions about it). However, even if *this* is the nature of *this* part of reality, why would it yield direct access to the nature of reality at large? This is the hard problem of mysticism. How could it be approached?

At the minimum, it appears that the insight that *this* is part of the fundamental nature of reality allows for the mystical-rational *inference* about the fundamental nature of the rest of reality, based on physicalistic premises. If consciousness is indeed a neural process in the brain, it is smoothly based on lower-level constituents in a process of weak emergence. The neural correlate of consciousness (i.e., consciousness as it is modeled by science) is constituted by the activity of individual neurons and synaptic processes, which are biochemical processes that can be reduced to how individual molecules and atoms interact. Ultimately, the neural correlate (i.e., consciousness) is nothing but a very big cloud of quarks interacting in a specific way, shaped by evolution. Thus, whatever the quarks are in themselves must be of a nature *continuous* with human consciousness (Figure 2). This can be taken to lead to a panpsychist or even idealistic view of the intrinsic nature of fundamental physical reality (Strawson, 2006; Jylkkä, 2016, 2022). This, however, is a rational argument, whereas the mystic claims to gain *direct access* to the fundamental nature. How could this be possible, if at all?

Stace (1961) acknowledges the hard problem (though not by this name) and proposes several possible solutions to it. One he considers as his “strongest argument” (p. 203) and it focuses on empty or pure consciousness that is void of content. For the sake of argument, we may presume that pure consciousness is possible, although this is disputed (see Jones and Gellman, 2022, §4). Stace’s argument can be called the *argument from no distinction*:

[I]f the undifferentiated unity [in a pure conscious event] is the pure unity of the individual self, then there is no *principium individuationis* on which can be based a distinction between one pure self and another. Therefore, we cannot stop at the individual ego, but are logically compelled to pass on to a Universal Self. I regard this as my strongest argument (p. 203).^22^

The argument can be reformulated as follows:

P1. In empty consciousness there is nothing to distinguish the “subject” from reality at large (i.e., there is no *principium individuationis*, PI)

P2. If nothing distinguishes between the subject and reality at large, then the subject is identical with reality at large (by way of identity of indiscernibles)

C. Thus, in empty consciousness the subject is one with reality at large.

In other words, if a pure conscious event has no PI, then nothing can distinguish it from the rest of reality. To illustrate, it could be held that reality consists of a substance that takes different forms (e.g., that of human consciousness), and when there is no specific form, there is only the underlying substance, which is unitary. The argument appears to be valid, but on closer examination there is a hidden premise between P1 and P2, which can be formulated as follows:

P1.5. If X is experienced to be empty (i.e., without PI), then X *is* empty (i.e., without PI).

This premise can be disputed: if an experience is experienced to lack properties, it may not follow that it truly lacks properties. From a naturalistic perspective it can be argued that any experience, even an empty one, is still a brain process and thus distinct from the rest of reality. For example, Metzinger (2020) argues that a pure conscious event is a Bayesian representation of tonic alertness, that is, a brain process clearly distinct from the rest of reality. It is unclear what it could mean, from the naturalistic perspective, for an experience to be truly without individuating properties. Arguably, the mystic would need to literally *become* a fundamental physical process, such as a quantum field. This appears to be quite unlikely, given that the brain is a wet and noisy environment where the quantum processes quickly decohere. On the other hand, this naturalistic objection to P1.5 could be criticized for presupposing the primacy of science in deciding whether a phenomenon has properties or not. If, by contrast to naturalism, it is experience that reveals the ultimate nature of reality, then it could be argued that an experience that lacks all phenomenal content *is* truly empty and, thus, lacks PI. In other words, the mystic might argue that P1.5 is indeed true. The argumentation in this case would become a *status quo* where the question pertains to the fundamental premise: whether to prioritize science or experience in the first place.

**Figure 3:**
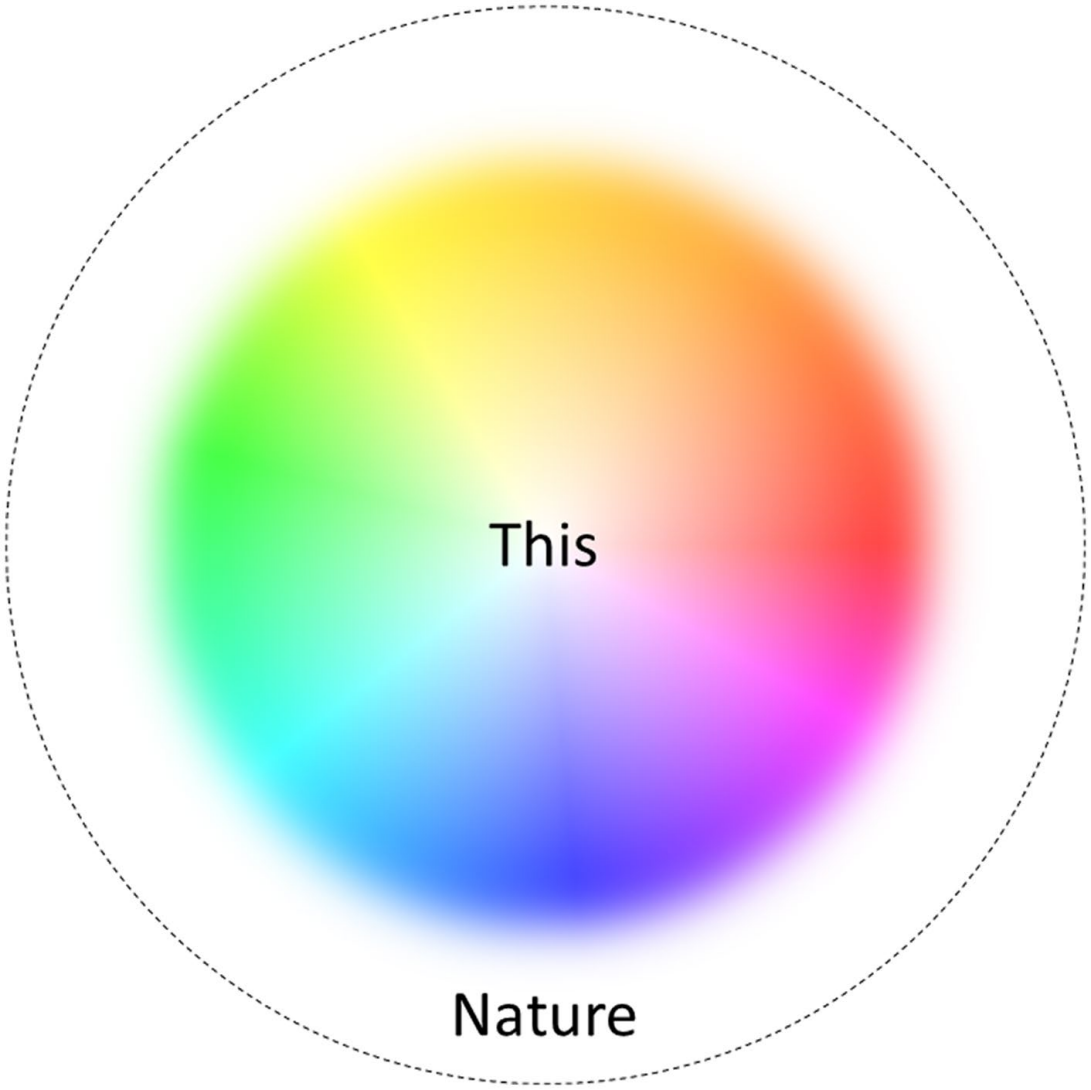
The whole of nature is represented as the white sphere, which can take different forms, represented as the colorful sphere. Human consciousness (*this*) is one such form, which we unitarily know through being it. Stace’s argument from no distinction entails that in a pure conscious event, the individuating forms of consciousness become dissolved, leading to direct contact with the reality at large: the colorful sphere becomes dissolved into the white one. However, even if such complete dissolution were impossible, psychedelic and mystical-type experiences can enable *this* to take more varied forms than is possible in non-altered consciousness, enabling an expansion of unitary knowledge.

Direct insight into the ultimate nature of reality could be considered as the Holy Grail of mystical experience and psychedelic-facilitated mysticism. It may be too ambitious, if by “reality” we mean *all* of reality. However, psychedelics and mystical-type experiences generally could provide more modest, but still substantial, epistemic benefits. Through being conscious, we gain a glimpse of the reality beyond our representations, and psychedelics could be considered to widen this view (Figure 3). Psychedelics literally expand consciousness through increasing the possible shapes it can take, enabling forms of consciousness that radically differ from everyday experience. Given that experiences are forms of reality, psychedelics enable us to unitarily know a wider range of forms of reality than is possible in non-altered consciousness. In other words, psychedelics expand unitary knowledge through enabling more varied forms of consciousness. Of particular interest is the case of empty or pure consciousness, which might be most radically different from everyday consciousness, showing the possibility of consciousness without any content or phenomenal form (assuming that such experiences in fact can take place; see Jones and Gellman, 2022). The possibility of such experience could be taken to show that consciousness does not require complex cognitive processes and could be a fundamental aspect of nature. Again, even if such experience would not reveal the ultimate nature of reality, it reveals how radically exotic forms of consciousness there can exist. This can be considered as a substantial epistemic benefit. The knowledge thusly gained can be considered as mystical-type, as it reveals something about the fundamental nature of reality: through widening the state-space of consciousness, it expands unitary knowledge.

In sum, it can be argued that psychedelic or mystical-type experiences can enable substantial increases unitary or mystical-type knowledge about the fundamental nature of reality, even if they could not reveal the fundamental nature of *all* of reality. Consciousness provides us with a glimpse into the fundamental nature of reality, and psychedelics could widen that view through expanding the forms that consciousness can take. Based on that glimpse, the mystic may intuit that that the ultimate nature of reality at large could be characterized as “some kind of light,” “universal love,” or even “God.” However, as is the case with most metaphysical conceptions of reality, there is no way of proving or disproving such ideas if they are conceptually consistent and compatible with what science tells us about the world. However, even if mystical-type conceptions of reality can be *inspired* by psychedelic or mystical-type experiences, I have argued that it is far from clear how the experience could *justify* any all-encompassing metaphysical views.

It is worth noting that, in addition to specifying how the mystical insight could reveal the nature of reality at large, the hard problem also involves elaborating the relationship between the mystical insight and conceptualizations of the insight. The problem is how a non-conceptual, direct insight can be translated into a conceptual representation. The problem is emphasized by the thesis that the ultimate nature of reality is its representation-independent nature: how could any representation capture the representation-independent nature of reality? Indeed, this is logically impossible. All representations are merely, as it were, images or reflections of reality formed in our minds, distinct from the reality in itself. This emphasizes the ineffability of the mystical insight, and indeed the ineffability of all experience. However, this does not entail that the mystical insight, or any experience for that matter, could not *inform* conceptual representations, or be the *object* of representation: from the naturalistic perspective, all experiences are causal processes that can interact with other processes, such as the cognitive system. The point is simply that when we reflect or represent an experience, the experience becomes “more” than it was prior to reflecting on it, and the representation fails to fully capture its nature as it was prior to reflection. More extensive discussion of this issue is beyond the scope of the present paper.

## 6 Conclusion

Some naturalist philosophers have argued that psychedelic mystical-type insights are incompatible with naturalism and therefore false. In contrast, I have argued that the Core thesis of mysticism, claiming that there is a unitary nature of reality beyond sense impressions, can be compatible with naturalism and its main component physicalism, when physicalism is conceived of in terms of Naturalistic Monism. Once we become aware of our (scientific) models of nature as merely models, we can see that there are many models of the same underlying reality. Indeed, psychedelics can bring about this insight through cognitive defusion, that is, by enabling us to see our models of reality *as* models, distinct from reality in itself. This can be considered as the main *negative* philosophical lesson of the mystical-type experience. Moreover, psychedelics can help us to see consciousness as it is in itself, through collapsing our reflective models of consciousness and enabling us to simply be aware in the here and now. Psychedelics also enable consciousness to take more varied forms, which broadens the glimpse we have of the fundamental nature of reality, insofar as experiences are part of reality in itself. I have argued that we can at least partially know the ultimate nature of reality through *being* part of nature, by consisting of the same fundamental entities and processes that physics models. It is at least *possible*, something not ruled out by our best knowledge of the world, that the ultimate nature of reality is as the mystic describes it. However, it is far from clear how mystical-type metaphysical conceptions about all of reality could be justified by mystical-type experiences.

In sum, the metaphysical claims of the mystic may be consistent with naturalism (i.e., the easy problem may be solvable), but the epistemological claims may not be (i.e., the hard problem might be unsolvable). However, even if the hard problem could not be solved, this does not render psychedelic-inspired metaphysical accounts about the ultimate nature of reality pointless. Eventually, *all* metaphysical conceptions of reality are beyond verification, and mystical-type conceptions are not an exception. Mystical-type insights may inspire or facilitate novel ideas about the nature of reality, but it is improbable that they could provide a shortcut to universal metaphysical truths. It is important to note that non-verifiability pertains even to the traditional naturalistic thesis that reality ultimately consists of dead matter that is void of consciousness or value – this thesis cannot be proved or disproved any more than the mystical-type thesis that God or universal consciousness is the ground of being. In any case, mystical-type experiences can constitute an *increase* in knowledge about the fundamental nature of reality, even if they could not *completely* reveal it.

I take it that the negative point of mystical-type insight – namely that we do not know the ultimate nature of reality, at least through observation, science and representation – is *the* most important philosophical implication of the experience, as it demonstrates the limits of our rational knowledge and fosters a sane and epistemically humble attitude toward the world. The realization of our conceptions of reality as *just* conceptions, artifacts of the human mind, can facilitate a more tolerant and less dogmatic attitude, where we do not conflate our words with objects. It is our shared human condition to be limited organisms in an unknown universe, trying to grasp it the best we can. Even if direct communion with the ultimate nature of all of reality were possible, that would be an ineffable, non-conceptual insight that does not vindicate dogmatic faith in any conceptual representations. Psychedelics amplify the importance of distinguishing our conceptions of reality from reality in itself. This point of caution applies to the mystic and naturalist alike – no one can claim that their conceptual representations capture the ultimate nature of reality.

## Data availability statement

The original contributions presented in the study are included in the article/supplementary material, further inquiries can be directed to the corresponding author.

## Author contributions

The author confirms being the sole contributor of this work and has approved it for publication.

## Footnotes

^1^Psychedelics are substances that can produce a specific kind of altered state of consciousness. They include so-called classical psychedelics such as lysergic acid diethylamide (LSD), psilocybin, and dimethyltryptamine (DMT) which act as agonists on the serotonin 5HT2A-receptor. Non-classical psychedelics, in turn, are a wide class of substances with varied neuropharmacological mechanisms and include, for example, cannabis, ketamine, ibogaine, and nitrous oxide. It could be argued that what makes a substance psychedelic is what kind of experience it facilitates, not *how* it does this.

^2^Psychedelics can facilitate a broad range of experiences and insights, not just mystical-type. Here I focus on mystical-type experiences because of their centrality in modern psychedelic research: for example, the intensity of mystical-type experience is a strong predictor of treatment outcomes in psychedelic-assisted therapy (Ko et al., 2022). Mystical-type experiences are typically conceptualized based on Stace’s (1961) work, which is the basis for the Mystical Experience Questionnaire (MEQ) (Barrett et al., 2015), commonly used in empirical psychedelic research. In this article, I focus on metaphysical insights, which are a central subcomponent of mystical-type experiences, but have been criticized by some authors as being delusional.

^3^I am borrowing the easy/hard problem dichotomy from the context of consciousness studies, as introduced by Chalmers (1995).

^4^Being outside time and space is a central aspect of the psychedelic experience, but unfortunately, I cannot go into it in the present article due to space limits. However, the illusoriness of space and time can be considered as compatible with, or even following from, certain accounts in physics (Rovelli, 2021).

^5^The quote can be found on several webpages, also in its alleged original form, scanned from a paper written with typewriter. Here I cite the Erowid site, on the assumption that the link is relatively stable.

^6^The mystical-type “theses” are conceptual representations of reality (e.g., the claim that the ultimate nature of reality is some type of sacred light), which are hypothetically based on, or facilitated by, the mystical-type insight. The insight itself, in turn, may be non-conceptual.

^7^By "scientific fact" I mean, broadly, a fact that is supported by substantial scientific evidence, or a fact entailed by a mature scientific theory. This does not entail that science could ever *prove* any statement as definitely true. Note that scientific and extra-scientific facts or theses are sometimes closely related. For example, it is an empirical question what is the neural correlate of consciousness, but an extra-scientific (or metaphysical) claim to say that the neural correlate is *identical* with consciousness.

^8^One might respond to these arguments that even if current science cannot capture the nature of phenomenal consciousness, it is possible that future science could. Such arguments are discussed and rejected in Chalmers (1996) and are not in the scope of the present article. In what follows, I argue that the gap is unbridgeable, because it is the difference between things in themselves and our representations of them.

^9^We may assume that organisms without brains can be phenomenally conscious. Some might even argue that computers could be conscious, but then again, *all* forms of reality can be considered as forms of consciousness. I strongly doubt that computers, being deeply different in physical layout than biological organisms, could manifest consciousness in any similar form as living organisms do. This claim rests on the premise that consciousness is *identical* with its physical correlate (the correlate *is* the experience seen “from the outside”), which implies that any two systems differing in physical layout cannot be strictly phenomenally identical (Jylkkä and Railo, 2019).

^10^Here I use the term “object” in an ontologically innocent sense, not implying anything about its nature: whether it is a thing, process, or something else.

^11^It can be argued that objects have no intrinsic nature that is independent of their relational and dispositional properties, but instead that the nature of an object is exhausted by its relational properties; the view is known as ontic structural realism (Ellis, 2001; Ladyman, 2023). This, however, does not imply that an object would not have an observer-independent nature, the observer being just one of all the entities that an object can interact with. On the typical reading, the term “intrinsic” is contrasted with “extrinsic” or “relational,” entailing that thoroughly relational entities like processes do not have intrinsic natures. However, “intrinsic nature of x” can also mean the observer- or modeler-independent nature of x, that is, the way x is independently of being observed or modeled by someone. I have previously called these two readings of intrinsic “strong” and “weak,” respectively (Jylkkä, preprint). Crucially, an object can lack a strong intrinsic nature (i.e., completely non-relational nature) if it is a process whose essence is exhausted by its relational properties, but it still possesses a weakly intrinsic nature in the sense that the process is the way it is independently of being observed and modeled. For example, water, conceived of as an interaction process between hydrogen and oxygen, could not exist independently of how hydrogen and oxygen interact, but it can exist and has whatever nature it has independently of being related to a modeler/observer who represents it as “water” or “H_2_O.” Note that this leads to Tarskian regress, although it is not problematic (Jylkkä, preprint).

^12^I have previously argued that the zombie-argument against physicalism dissolves once we notice that the term “neural correlate of consciousness” refers rigidly to consciousness (Jylkkä, 2013). Note that it could be disputed whether “consciousness” or “neural correlate of consciousness” are natural kind terms. For example, Rey (1983) and other eliminativist philosophers have presented arguments that “consciousness” refers to a diverse set of processes instead of a natural kind. However, such diversity *per se* need not entail that the referent is not a natural kind; it could be, for example, a homeostatic cluster kind. For more discussion on this topic, see Bayne and Shea (2020).

^13^This is illustrated by a dramatic experiment on brain damaged patients, thought to be in a vegetative state, i.e., lacking consciousness. In an experiment, one such patient was asked questions and was instructed to imagine playing tennis if the answer is “yes,” and to imagine navigating in their home if the answer was “no.” These mental imagings could be detected as distinct activation patterns in fMRI. To the researchers’ surprise, the patient could correctly answer the questions (Owen et al., 2006).

^14^For example, when a subject X imagines red in fMRI, it is the experience of X that underlies the NCC_red_ observations made by another subject Y, that is, the neuroscientist looking at the fMRI scans.

^15^Although I rely on the duality between representation and the represented to make my point, the relationship between the two is complex. For example, in visual perception I directly interact with the perceived object: the photons reflected from the cup affect my retina and visual cortex, and microsaccades in my eyes continually modify the way the cup is reflected on the retina. Moreover, I can grab the cup and take a sip of coffee, which inhibits my adenosine receptors, making me more alert. This is a constant interaction process, and I am part of it; it is *this*. In other words, there is no clear distinction between representation and object. However, the term “representation” can be used to emphasize that the perception of the cup is a brain-dependent process, which is shaped by our nervous system, in addition to the cup.

^16^Unitary knowledge is similar to Forman’s (1993) “knowledge by identity,” but for him the notion applies merely to pure conscious events that lack content. By contrast, I take it that unitary knowledge is present in *all* experience.

^17^It could be objected that Pollan’s experience is nevertheless *about* the music. However, from the subjective perspective it is not about anything; it is “about” the music only when it is so *modeled* by another subject, or Pollan himself at a later time. It is the subjective perspective which is crucial here, as only first-person experience can constitute unitary knowledge. It makes no difference whether the experience is realized solely inside the brain, or in interaction between the brain and some external processes.

^18^By “pure awareness” I do not mean consciousness void of sensory contents or “forms,” but instead awareness that is not *of* or *about* anything. It is awareness which knows itself without reflection, without duality.

^19^For a more extensive discussion of *this*, see Jylkkä, (2022). See also Forman’s (1993) discussion of knowledge by identity, which is very similar to my notion of unitary knowledge, although Forman considers knowledge by identity to be present only in pure conscious events.

^20^*Cf.* Harris (2006, p. 6). Cognitive defusion is a central notion in commitment and acceptance therapy (ACT) but it stems from the wider mindfulness literature and can be applied outside the clinical context.

^21^For an excellent introduction to *this* from a Zen perspective, see Watts (1957).

^22^The argument appears in different formulations through Stace’s book; see, e.g., pp. 110–111.

## References

[ref1] Allais L. (2015). Manifest reality: Kant’s idealism and his realism. New York: Oxford UP.

[ref2] Audi R. (1995). Cambridge dictionary of philosophy. Cambridge: Cambridge UP.

[ref3] Barrett F. S. Johnson M. W. Griffiths R. R. (2015). Validation of the revised mystical experience questionnaire in experimental sessions with psilocybin. J. Psychopharmacol. 29, 1182–1190. doi: 10.1177/0269881115609019, PMID: 26442957 PMC5203697

[ref4] Bayne T. Shea N. (2020). Consciousness, concepts, and natural kinds. Philos. Top. 48, 65–83. doi: 10.5840/philtopics20204814

[ref5] Blofeld J. (1958). The Zen teachings of Huang Po: On the transmission of mind. New York: Grove Press.

[ref6] Carhart-Harris R. L. Friston K. J. (2019). REBUS and the anarchic brain: toward a unified model of the brain action of psychedelics. Pharmacol. Rev. 71, 316–344. doi: 10.1124/pr.118.017160, PMID: 31221820 PMC6588209

[ref7] Chalmers D. (1995). Facing up to the problem of consciousness. J. Conscious. Stud. 2, 200–219.

[ref8] Chalmers D. (1996). The conscious mind: In search of a fundamental theory. Oxford: Oxford UP.

[ref9] Chalmers D. (2019). “Idealism and the mind-body problem” in The Routledge handbook of Panpsychism. ed. Seager W. (New York: Routledge), 353–373.

[ref10] Dennett D. (1991). Consciousness explained. Boston: Little, Brown and Co.

[ref11] Eddington A. (1929). The nature of the physical world. Cambridge: Cambridge UP.

[ref12] Ellis B. (2001). Scientific Essentialism. Cambridge: Cambridge UP.

[ref13] Forman R. K. C. (1993). Mystical knowledge: knowledge by identity. J. Am. Acad. Relig. LXI, 705–738. doi: 10.1093/jaarel/LXI.4.705

[ref14] Frankish K. (2017). Illusionism as a theory of consciousness. Exeter: Imprint Academic.

[ref15] Friston K. (2010). The free-energy principle: a unified brain theory? Nat Rev Neurosci 11, 127–138. doi: 10.1038/nrn2787 20068583

[ref16] Goff P. (2017). Consciousness and fundamental reality. Oxford: Oxford UP.

[ref17] Harris R. (2006). Embracing your demons: An overview of acceptance and commitment therapy. Psychotherapy Australia 12, 2–8.

[ref18] Hiddleston E. (2019). Dispositional and categorical properties, and Russellian monism. Philos. Stud. 176, 65–92. doi: 10.1007/s11098-017-1006-2

[ref19] Hietanen J. Turunen P. Hirvonen I. Karisto J. Pättiniemi I. Saarinen H. (2020). How not to criticise scientism. Metaphilosophy 51, 522–547. doi: 10.1111/meta.12443

[ref20] Hohwy J. (2013). The predictive mind. Oxford: Oxford UP

[ref21] Howell R. (2015). The Russellian Monist’s problems with mental causation. Philos. Q. 65, 22–39. doi: 10.1093/pq/pqu058

[ref22] Huxley A. (1954). Doors of perception. New York: Harper & Row.

[ref23] Jackson F. (1986). What Mary Didn’t know. J. Philos. 83, 291–295. doi: 10.2307/2026143

[ref24] James W. (1902). The varieties of religious experience: A study in human nature. London: Longmans, Green and Co

[ref25] Jones R. Gellman J. (2022). Mysticism. Stanford encyclopedia of philosophy. Available at: https://plato.stanford.edu/entries/mysticism/

[ref26] Jylkkä J. (2013). Natural concepts, phenomenal concepts, and the conceivability argument. Erkenntnis 78, 647–663. doi: 10.1007/s10670-012-9368-5

[ref27] Jylkkä J. (2016). Mind as an intrinsic property of matter. Philos. Investig. 39, 15–37. doi: 10.1111/phin.12100

[ref28] Jylkkä J. (2022). “Mary on acid: experiences of unity and the epistemic gap” in Philosophy and psychedelics. eds. Hauskeller C. Sjöstedt-H P. (London: Bloomsbury Academic)

[ref29] Jylkkä J. Railo H. (2019). Consciousness as a concrete physical phenomenon. Conscious. Cogn. 74:102779. doi: 10.1016/j.concog.2019.102779, PMID: 31295656

[ref30] Kind A. (2015). “Pessimism about Russellian monism” in Consciousness in the physical world: Perspectives on Russellian monism. eds. Alter T. Nagasawa Y. (Oxford: Oxford UP), 401–421.

[ref31] Ko K. Knight G. Rucker J. J. Cleare A. J. (2022). Psychedelics, mystical experience, and therapeutic efficacy: a systematic review. Front Psychiatry 13:917199. doi: 10.3389/fpsyt.2022.917199 35923458 PMC9340494

[ref32] Kripke S. (1980). Naming and necessity. Harvard: Harvard UP.

[ref33] Ladyman J. (2023). Structural realism. Stanford encyclopedia of philosophy. Retrieved from https://plato.stanford.edu/entries/structural-realism/

[ref34] Langton R. (2001). Kantian humility: Our ignorance of things in themselves. Oxford: Oxford UP

[ref35] Letheby C. (2021). Philosophy of psychedelics. Oxford: Oxford UP

[ref36] Letheby C. Gerrans P. (2017). Self unbound: ego dissolution in psychedelic experience. Neurosci. Consciousness, 1–11. doi: 10.1093/NC/NIX016, PMID: 30042848 PMC6007152

[ref37] Levine J. (1983). Materialism and qualia: the explanatory gap. Pac. Philos. Q. 64, 354–361. doi: 10.1111/j.1468-0114.1983.tb00207.x

[ref38] Lutz M. Lenman J. (2018). Moral Naturalism. Stanford encyclopedia of philosophy. Available at: https://plato.stanford.edu/entries/naturalism-moral/

[ref39] Masters R. A. (2010). Spiritual bypassing: When spirituality disconnects us from what really matters. Berkeley, CA: North Atlantic Books.

[ref40] Merriam-Webster. (n.d.). Mystical. Available at: https://www.merriam-webster.com/dictionary/mystical

[ref41] Metzinger T. (2020). Minimal phenomenal experience: meditation, tonic alertness, and the phenomenology of “pure” consciousness. Philosophy Mind Sci 1, 1–44. doi: 10.33735/phimisci.2020.I.46

[ref42] Montero B. (2015). “Russellian physicalism” in Consciousness in the physical world. eds. Alter T. Nagasawa Y. (Oxford: Oxford UP), 209–223.

[ref43] Nagel T. (1974). What is it like to be a bat?. Philosophical Review 4, 435–450. doi: 10.2307/2183914

[ref44] Odin S. (2022). “The unconscious in zen and psychedelic experience” in Philosophy and psychedelics. eds. Hauskeller C. Sjöstedt-H P. (London: Bloomsbury Academic)

[ref45] Orr M. (2006). What is a scientific world view, and how does it bear on the interplay of science and religion? Zygon 41, 435–444. doi: 10.1111/J.1467-9744.2005.00748.X

[ref46] Owen A. M. Coleman M. R. Boly M. Davis M. H. Laureys S. Pickard J. D. (2006). Detecting awareness in the vegetative state. Science 313:1402. doi: 10.1126/science.1130197 16959998

[ref47] Pahnke W. N. (1964). First impressions of first LSD experience. Available at: erowid.org/exp/103042

[ref48] Papineau D. (2020). Naturalism. Stanford encyclopedia of philosophy. Available at: https://plato.stanford.edu/entries/naturalism/

[ref49] Pollan M. (2018). How to change your mind. New York: Penguin Books.

[ref50] Putnam H. (1975). The meaning of “meaning.”. Minn. Stud. Philos. Sci. 7, 131–193. doi: 10.1017/CBO9780511625251.014

[ref51] Revonsuo A. (2006). Inner presence: Consciousness as a biological phenomenon. Massachusetts: MIT Press.

[ref52] Rey G. (1983). “A reason for doubting the existence of consciousness” in Consciousness and self-regulation. eds. Davidson R. Schwartz G. Shapiro D. (New York: Plenum), 1–39.

[ref53] Richards W. A. (2016). Sacred knowledge: Psychedelics and religious experiences. Columbia: Columbia UP.

[ref54] Rosen G. (2017). “Metaphysical relations in metaethics” in The Routledge Handbook of Metaethics. eds. McPherson T. Plunkett D. (New York: Routledge), 151–169.

[ref55] Rovelli C. (2021). Helgoland. London: Allen Lane.

[ref56] Russell B. (1910). Knowledge by acquaintance and knowledge by description. Proc. Aristot. Soc. 11, 108–128.

[ref57] Russell B. (1927). The analysis of matter. London: Kegan Paul.

[ref58] Sanders J. W. Zijlmans J. (2021). Moving past mysticism in psychedelic science. ACS Pharmacol. Transl. Sci 4, 1253–1255. doi: 10.1021/acsptsci.1c00097, PMID: 34151217 PMC8205234

[ref59] Sjöstedt-H P. (2022). “The white sun of substance: Spinozism and the psychedelic amor Dei intellectualis” in Philosophy and psychedelics. eds. Hauskeller C. Sjöstedt-H P. (London: Bloomsbury Academic)

[ref60] Smith H. (2000). Cleansing the doors of perception: The religious significance of Entheogenic plants and chemicals. Boulder: Sentient Publications.

[ref61] Stace W. T. (1961). Mysticism and philosophy. London: MacMillan.

[ref62] Stone J. A. (2012). Spirituality for naturalists. Zygon 47, 481–500. doi: 10.1111/j.1467-9744.2012.01279.x

[ref63] Strawson G. (2003). “Real materialism” in Chomsky and his critics. eds. Louise M. A. Hornstein N. (Malden, MA: Blackwell), 49–88.

[ref64] Strawson G. (2006). Realistic monism: why physicalism entails Panpsychism. J. Conscious. Stud. 13, 53–74.

[ref65] Strawson G. (2019). “The Mary-go-round” in The knowledge argument. ed S. Coleman (Cambridge: Cambridge UP), 118–140.

[ref66] Swanson L. R. (2016). The predictive processing paradigm has roots in Kant. Front. Syst. Neurosci. 10:79. doi: 10.3389/fnsys.2016.00079, PMID: 27777555 PMC5056171

[ref67] Tononi G. Boly M. Massimini M. Koch C. (2016). Integrated information theory: from consciousness to its physical substrate. Nature reviews neuroscience. 17, 450–461. doi: 10.1038/nrn.2016.44 27225071

[ref68] Urmson J. O. Ree J. (1989). Concise encyclopedia of Western philosophy and philosophers. London: Unwin Hyman.

[ref69] Watts A. (1957). The way of Zen. New York: Pantheon Books.

[ref70] Watts A. (1962). Joyous cosmology. New York: Pantheon Books.

[ref71] Zahavi D. (2018). Brain, mind, world: predictive coding, neo-Kantianism, and transcendental idealism. Husserl Stud. 34, 47–61. doi: 10.1007/S10743-017-9218-Z PMC5723412 29267407

